# The pathophysiology of negative symptoms of schizophrenia: main hypotheses and open challenges

**DOI:** 10.1192/bjp.2023.63

**Published:** 2023-07

**Authors:** Silvana Galderisi, Stefan Kaiser

**Affiliations:** Department of Mental and Physical Health and Preventive Medicine, University of Campania Luigi Vanvitelli, Naples, Italy; Department of Psychiatry, Geneva University Hospitals, Geneva, Switzerland

**Keywords:** Negative symptoms, schizophrenia, pathophysiology, neurobiological research, hypofrontality

## Abstract

Important developments in the conceptualisation and classification of negative symptoms have contributed to refining hypotheses on their pathophysiology. The uptake of recent progress is still only partial and the whole field might make a leap forward once relevant studies fully make use of assessment tools based on current conceptualisations.

Although an increasing number of studies have investigated the pathophysiology of negative symptoms, a clear neuroscience-based model has still to emerge. This is not surprising given the dearth of research on pathophysiology taking into account current developments in the conceptualisation and classification of negative symptoms.

## Differentiating negative symptoms

In fact, most neurobiological research has investigated negative symptoms as a homogeneous psychopathological domain, often searching for correlates of a total score that sums very heterogeneous symptom domains and is therefore difficult to interpret. Therefore, the inconsistent findings on the pathophysiology of these symptoms are not surprising. Two important distinctions in the assessment of negative symptoms are now well-established and need to be taken into account in research on pathophysiology.

First, negative symptoms consist of five domains that can be grouped into the experiential dimension (avolition, asociality, anhedonia) and the expressive dimension (alogia, blunted affect). There is now a consensus that a differentiated assessment of these dimensions, and preferably of the five domains, should be attempted in any study targeting negative symptoms, but the exact level of granularity required remains a matter of debate.

Second, primary negative symptoms should be differentiated from secondary negative symptoms due to positive symptoms, medication side-effects and depression. Unfortunately, the clinical trials literature often ignores this issue, and the same may be said of the literature on pathophysiology.

Thus, today clear concepts exist for both the distinction of different negative symptom domains and the distinction of primary versus secondary negative symptoms, but research on pathophysiology and treatment has not yet fully embraced these concepts.

## Functional and structural neuroimaging

A few examples from studies focusing on associations shown by negative symptoms with neuroimaging abnormalities may substantiate these considerations.

The striking resemblance of negative symptoms of schizophrenia to apathy observed after frontal lobe damage led to a focus on the frontal lobes in research on neurobiological underpinnings of negative symptoms. However, several structural magnetic resonance imaging (MRI) studies failed to find a relationship between negative symptoms and total frontal area or volume in schizophrenia.^1^ There are interesting findings on associations of negative symptoms with specific frontal regions, but they lack consistency. Meta-analytic findings from the ENIGMA consortium suggest an association of total negative symptoms with cortical thinning in the medial orbitofrontal cortex.^2^ However, it is also clear that the meta-analytic approach is limited by the degree of differentiated assessment in the individual studies.

Findings from functional imaging studies raised optimistic expectations: in particular, positron emission tomography (PET) and early functional MRI (fMRI) studies pointed to hypofrontality as the neurobiological substrate of negative symptoms and, more often, reported an association of negative symptoms with a dysfunction of the dorsolateral prefrontal cortex (DLPFC). However, a meta-analysis focusing on fMRI studies found that negative symptoms were not associated with a dysfunction of the DLPFC but with a hypoactivation of the ventrolateral prefrontal cortex (VLPFC) and ventral striatum,^3^ but for each of the latter regions only two studies were available at the time and the results have therefore to be interpreted with caution.

Although evidence for the association between negative symptoms and hypoactivation of the VLPFC remains sparse, more consistent findings have emerged with respect to the ventral striatum. In an important meta-analysis, Radua et al included 23 studies investigating reward anticipation and confirmed the association between negative symptoms and hypoactivation of the left ventral striatum.^4^ Some studies have suggested that this association may be specific to experiential negative symptoms, but this finding remains to be confirmed in larger studies. Unfortunately, in many of these studies a clear distinction between primary and secondary negative symptoms has not been made. Furthermore, hypoactivation of the ventral striatum also correlates with positive symptoms and tends to normalise, especially in the patients with the most pronounced treatment effect on the positive symptoms, and therefore ‘pseudospecificity’ may be an issue.

The research discussed above has focused more on brain circuits that are potentially relevant for experiential negative symptoms, and the pathophysiology of expressive negative symptoms has been much less explored. The paper by Canal Rivero et al (this issue) provides a welcome contribution to the topic and has several important strengths, including the large number of study participants in their first episode of psychosis, the multiple time points, the 10-year follow-up, the multimodal characterisation of the participants and the sophisticated statistical analysis. Study participants with increasing expressivity presented greater cortical thinning between 3 and 10 years after the onset of the psychotic disorder in four particular brain regions (i.e. caudal middle frontal, pars triangularis, rostral middle frontal and superior frontal). At odds with previous studies, no brain alteration was found in association with the experiential dimension. The authors hypothesise that the negative finding is due to the longitudinal design of the study. However, it should be noted that in their study they used a scale that focuses on behavioural aspects of avolition (a key component of the experiential factor) and may neglect internal experience/drive. The assessment of both behaviour and internal experience is required by current conceptualisations of avolition.

## Neurotransmitters and genetics

Although many treatment approaches in psychiatry have been fortuitous and at least initially not based on our understanding of neural circuits, this approach has found its limits in the treatment of negative symptoms. We believe that in the future an understanding of disturbed neural circuits will be necessary to make progress. In this respect, structural and functional neuroimaging provide important contributions to understanding the pathophysiology of negative symptoms, but an understanding of the related neurotransmitter changes will be needed to use these findings for developing neuroscience-based treatment approaches. In the study by Canal Rivero et al a new approach of mapping structural findings to a PET-based atlas is employed, suggesting that the expressivity dimension associated cortical thinning is found in cortical regions with lowest receptor density. This is certainly a preliminary finding, but this type of mapping opens up new avenues for understanding the pathophysiology of negative symptoms.

A more direct approach to studying molecular alterations underlying psychopathology is, of course, based on PET imaging in patients. Here, recent studies have started to converge on an association of negative symptoms with reduced striatal dopamine synthesis capacity measured using ^18^F-DOPA PET, which could provide an interesting link with the findings of reduced striatal activation during reward anticipation. These findings may emphasise the need to find alternatives to dopamine blocking agents for the treatment of negative symptoms, but again a more differentiated approach to negative symptom assessment would be very useful.

We would also like to mention that researchers have recently developed a strong interest in the genetic basis of symptom dimensions. Several studies have found familial aggregation of negative symptoms, suggesting a genetic basis for this symptom dimension. In an important paper Ahangari and colleagues (this issue) show that the polygenic risk score for schizophrenia is associated with negative/disorganised symptoms in people with schizophrenia and with negative symptoms in non-psychotic relatives from multiplex families with schizophrenia. Their findings further reinforce the notion that the negative symptom dimension may have a strong genetic basis. They also highlight the challenges for a differentiated assessment of negative symptoms in studies on genetics that commonly include larger samples than neuroimaging studies and focus more on a lifetime than on a current symptoms profile. Nevertheless, the lifetime focus could converge with primary negative symptoms and the exploration of the genetic basis of experiential versus expressive symptoms could be of great interest.

## How to move forward?

In the past two decades important developments in the conceptualisation and classification of negative symptoms have contributed to refining hypotheses and designing studies to test them. Thanks to this progress, research in the field can now benefit from a clear definition of which symptoms should be regarded as negative symptoms, as well as from criteria for identifying at least the most common sources of secondary negative symptoms and from assessment tools that reflect current consensus on what should be evaluated and how.^5^ The uptake of recent progress in this direction is still only partial. We strongly believe that research on the pathophysiology of negative symptoms will make a leap forward once studies using structural, functional and molecular neuroimaging, as well as genetics, begin to fully make use of assessment tools reflecting current conceptualisations and classifications of negative symptoms.

## Data Availability

Data availability is not applicable to this article as no new data were created or analysed in this study.
